# Echocardiographic Global Longitudinal Strain Is Associated With Myocardial Fibrosis and Predicts Outcomes in Aortic Stenosis

**DOI:** 10.3389/fcvm.2021.750016

**Published:** 2021-11-10

**Authors:** Thu-Thao Le, Weiting Huang, Gurpreet K. Singh, Desiree-Faye Toh, See Hooi Ewe, Hak Chaw Tang, Germaine Loo, Jennifer A. Bryant, Briana Ang, Edgar Lik-Wui Tay, Wern Miin Soo, James Wei-Luen Yip, Yen Yee Oon, Lingli Gong, Josephien B. Lunaria, Quek Wei Yong, Evelyn Min Lee, Poh Shuan Daniel Yeo, Siang Chew Chai, Ping Ping Goh, Lee Fong Ling, Hean Yee Ong, Arthur Mark Richards, Victoria Delgado, Jeroen J. Bax, Zee Pin Ding, Lieng-Hsi Ling, Calvin W. L. Chin

**Affiliations:** ^1^National Heart Research Institute Singapore, National Heart Center Singapore, Singapore, Singapore; ^2^Cardiovascular ACP, Duke-NUS Medical School Singapore, Singapore, Singapore; ^3^Department of Cardiology, National Heart Center Singapore, Singapore, Singapore; ^4^Department of Cardiology, Heart and Lung Centre, Leiden University, Leiden, Netherlands; ^5^Department of Cardiology, National University Heart Center Singapore, Singapore, Singapore; ^6^Asian Heart and Vascular Center, Mount Elizabeth Novena Hospital, Singapore, Singapore; ^7^Department of Cardiology, Sarawak Heart Centre, Sarawak, Kota Samarahan, Malaysia; ^8^Yong Loo Lin School of Medicine, National University of Singapore, Singapore, Singapore; ^9^Department of Cardiology, Tan Tock Seng Hospital, Singapore, Singapore; ^10^Apex Heart Clinic, Gleneagles Hospital, Singapore, Singapore; ^11^Department of Cardiology, Changi General Hospital, Singapore, Singapore; ^12^Department of Cardiology, Khoo Teck Puat Hospital, Singapore, Singapore; ^13^Cardiovascular Research Institute, National University Health System, Singapore, Singapore; ^14^Christchurch Heart Institute, University of Otago, Christchurch, Christchurch, New Zealand

**Keywords:** myocardial fibrosis, cardiovascular magnetic resonance, global longitudinal strain (GLS), aortic stenosis (AS), hypertensive heart disease (HHD)

## Abstract

**Aims:** Left ventricular ejection fraction is the conventional measure used to guide heart failure management, regardless of underlying etiology. Left ventricular global longitudinal strain (LV-GLS) by speckle tracking echocardiography (STE) is a more sensitive measure of intrinsic myocardial function. We aim to establish LV-GLS as a marker of replacement myocardial fibrosis on cardiovascular magnetic resonance (CMR) and validate the prognostic value of LV-GLS thresholds associated with fibrosis.

**Methods and results:** LV-GLS thresholds of replacement fibrosis were established in the derivation cohort: 151 patients (57 ± 10 years; 58% males) with hypertension who underwent STE to measure LV-GLS and CMR. Prognostic value of the thresholds was validated in a separate outcome cohort: 261 patients with moderate-severe aortic stenosis (AS; 71 ± 12 years; 58% males; NYHA functional class I–II) and preserved LVEF ≥50%. Primary outcome was a composite of cardiovascular mortality, heart failure hospitalization, and myocardial infarction. In the derivation cohort, LV-GLS demonstrated good discrimination (c-statistics 0.74 [0.66–0.83]; *P* < 0.001) and calibration (Hosmer-Lemeshow χ^2^ = 6.37; *P* = 0.605) for replacement fibrosis. In the outcome cohort, 47 events occurred over 16 [3.3, 42.2] months. Patients with LV-GLS > −15.0% (corresponding to 95% specificity to rule-in myocardial fibrosis) had the worst outcomes compared to patients with LV-GLS < −21.0% (corresponding to 95% sensitivity to rule-out myocardial fibrosis) and those between −21.0 and −15.0% (log-rank *P* < 0.001). LV-GLS offered independent prognostic value over clinical variables, AS severity and echocardiographic LV mass and E/e′.

**Conclusion:** LV-GLS thresholds associated with replacement myocardial fibrosis is a novel approach to risk-stratify patients with AS and preserved LVEF.

## Graphical Abstract

Thresholds of echocardiographic left ventricular global longitudinal strain (GLS) that are associated with replacement myocardial fibrosis risk-stratify patients with aortic stenosis and preserved left ventricular ejection fraction. Some images in the figure were created with BioRender.com. 
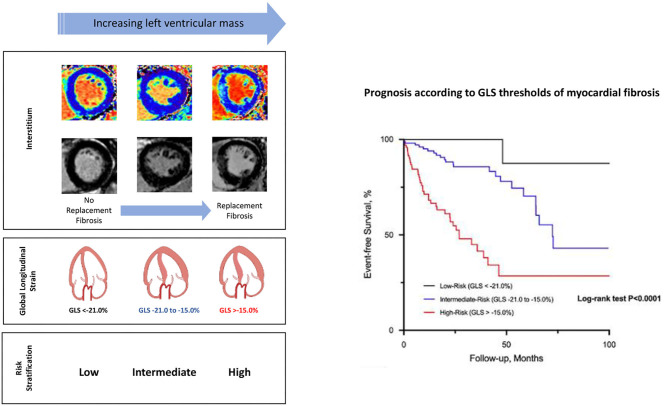

## Introduction

Myocardial fibrosis is one of the hallmarks of a failing heart (1). In non-ischemic etiologies, the accumulation of collagen in the interstitium is progressive: from reactive interstitial (microscopic) fibrosis in the early stages to replacement (macroscopic) fibrosis in more advanced stages of heart failure (2, 3). These changes in collagen composition result in increased myocardial stiffness, left ventricular (LV) diastolic and systolic dysfunction (4, 5). Therefore, there is increasing clinical interest in assessing the myocardial interstitium as a marker of decompensation and a potential therapeutic target in heart failure (6–8).

Regardless of etiology, impaired LV ejection fraction (LVEF) is commonly used as a measure of global LV systolic dysfunction to guide heart failure management. However, LVEF is a late marker of cardiac decompensation. Global longitudinal strain (GLS) using speckle tracking echocardiography is more sensitive in detecting early LV dysfunction and correlates with myocardial fibrosis on histology (9). Despite the potential utility and accuracy in detecting fibrosis, LV GLS thresholds associated with myocardial fibrosis as markers of cardiac decompensation and prognosis have not been well-investigated (9).

In Stage B heart failure (structural heart abnormalities without signs and symptoms of heart failure), adverse myocardial remodeling in left ventricular hypertrophy (LVH) conditions such as hypertensive heart disease and aortic stenosis (AS) is accompanied by myocardial fibrosis that is mediated in part by myocardial injury/ischemia, neurohormones and the renin-angiotensin-aldosterone system (10, 11). In these conditions, we have previously demonstrated that markers of myocardial fibrosis and advanced left ventricular hypertrophy (LVH) predicted worse outcomes (12–17).

Independent of underlying etiology, LV GLS reflects the functional consequences of myocardial fibrosis. In this study, we aim to examine the mechanistic association between LV GLS and the myocardium; and establish optimal LV GLS thresholds associated with myocardial fibrosis on cardiovascular magnetic resonance (CMR) in a cohort of hypertensive patients who have undergone both CMR and echocardiography. Subsequently, we will validate the prognostic value of these LV GLS thresholds in a separate cohort of asymptomatic/minimally symptomatic (NYHA functional class I–II) patients with moderate to severe AS and preserved LVEF.

## Methods

### Study Populations

Two cohorts of patients were used in this study. The derivation cohort consisted of patients from an on-going hypertension study (REMODEL, clinicaltrials.gov identifier: NCT02670031) at the National Heart Center Singapore (NHCS). The aim of the REMODEL trial was to examine the role of CMR in patients with hypertension. The inclusion and exclusion criteria were published previously (16, 17). Patients without incidental myocardial infarction, other cardiomyopathies (hypertrophic, dilated and infiltrative) and regional wall motion abnormalities on CMR were recruited for speckle-tracking echocardiography that was performed within 1 month of CMR.

The outcome cohort consisted of asymptomatic/minimally symptomatic (NYHA functional class I–II) patients with moderate and severe AS (peak aortic velocity, Vm ≥ 3 m/s or aortic valve area, AVA ≤ 1.2 cm^2^) and LVEF ≥50% from a multicenter Singapore study coordinated by the National University Heart Center Singapore (NUHCS) as well as an ongoing AS registry at the Leiden University Medical Center (LUMC). Patients with regional wall motion abnormalities on echocardiography were excluded. Patients in the Singapore cohort were prospectively recruited in September 2010 and followed until March 2021. Patients in the Netherlands cohort were followed up until October 2018.

The study was conducted in accordance with the Declaration of Helsinki and was approved by the individual research ethics committee. Written informed consent was obtained for patients in the Singapore cohorts. Consent was waived in the LUMC patients because of the retrospective nature of the study.

### Cardiovascular Magnetic Resonance Imaging and Analysis

All patients in the derivation cohort had CMR (Siemens Aera 1.5T, Siemens Healthineers, Erlangen, Germany). Balanced steady-state free precession cine images were acquired in the standard long-axis (two-, three-, and four-chamber) and short-axis views (acquired voxel size: 1.6 × 1.3 × 8.0 mm slice thickness; 2 mm gap; 30 phases per cardiac cycle). Myocardial fibrosis was assessed using two approaches: late gadolinium-enhanced (LGE) imaging for replacement myocardial fibrosis and myocardial T1 mapping for reactive interstitial myocardial fibrosis. LGE imaging was performed ~8 min after administration of 0.1 mmol/kg of gadobutrol (Gadovist; Bayer Pharma AG, Germany). An inversion-recovery fast gradient echo sequence was used, and the inversion time was optimized to achieve appropriate nulling of the myocardium. Myocardial T1 mapping based on the Modified Look-Locker inversion-recovery sequence (heartbeat acquisition scheme of 5(3)3 and 4(1)3(1)2 for native and post-contrast myocardial T1, respectively). Replacement myocardial fibrosis on LGE was assessed qualitatively. Extracellular volume fraction (ECV) was estimated from the native and 15-min post-contrast T1 map, analyzed using the T1 mapping module (CVI42, Circle Cardiovascular Imaging, Calgary, Canada) (18, 19). Hematocrit for ECV calculation was sampled on the day of CMR.

Cardiac volumes, function and LV mass were analyzed using the CVI42 software (Circle Cardiovascular Imaging, Calgary, Canada) at the National Heart Research Institute of Singapore (NHRIS) CMR Core Laboratory. Image analyses were performed by trained individuals using standardized protocols (20).

### Echocardiography Protocol

LV GLS assessment by two-dimensional speckle tracking echocardiography was performed at the individual centers. LV GLS was analyzed by post-processing triplicate apical LV images (two-, three- and four-chamber views) using EchoPAC software (GE Healthcare, Horten, Norway). Myocardial tracking was carefully verified and manually adjusted, if needed. Width of the region of interest was adjusted to include the entire thickness of the LV myocardium.

AS severity (aortic valve area, AVA; Vm; mean pressure gradient, MPG) in the outcome cohort was assessed according to the American Society of Echocardiography/European Association of Cardiovascular Imaging guidelines (21). The ratio of early diastolic mitral inflow velocity (E) and averaged LV medial and lateral annular velocities (e′) was used to estimate LV filling pressures (E/e′) (22). LVEF was assessed using the modified biplane Simpson method (average of the 2- and 4-chambers) (23).

### Clinical Outcomes

The primary outcome was a composite of first major adverse cardiovascular events: cardiovascular mortality, heart failure hospitalization and myocardial infarction. Secondary outcome was a composite of heart failure hospitalization, myocardial infarction, aortic valve replacement (AVR), and death from any cause. Events were censored at the time of AVR or last patient contact if no events were experienced.

### Statistical Analysis

Data were reported as percentages for categorical variables and mean ± standard deviation or median (interquartile range) for continuous variables as appropriate. The distribution of all continuous variables was assessed using the Shapiro–Wilk test. Depending on the normality of the distribution, parametric Student's *t*-test or non-parametric Kruskal–Wallis test were used to compare groups of continuous data. Categorial data were compared using the χ^2^ test. Statistical analyses were performed using SPSS version 23 (IBM Corp., Armonk, NY, USA). A two-sided *P* < 0.05 was considered statistically significant.

In the derivation cohort, multivariable logistic regression (forward step-wise selection method) was used to establish relevant determinants of myocardial fibrosis: age, sex, systolic blood pressure and LV GLS. The diagnostic performance of the model was assessed using the *c* statistic for discrimination (area under the receiver operating characteristic curve; AUC) and the Hosmer-Lemeshow goodness of fit for calibration. Optimal LV GLS thresholds defined as ≥95% specificity and ≥95% sensitivity for myocardial fibrosis on CMR were established from the AUC. These values would define the risk categories of patients in the outcome cohort.

In the outcome cohort, LV GLS values less than the sensitive threshold defined a low-risk category. Conversely, LV GLS greater than the specific threshold for myocardial fibrosis was categorized as high-risk. LV GLS values between the sensitive and specific thresholds were considered as intermediate-risk. Time-to-first major event survival curves associated with the different risk categories were estimated using the Kaplan–Meier method and compared with the log-rank test. The Cox regression model was used to adjust for potential confounders (age, sex, AS severity, LVEF and E/e′ ratio).

## Results

The derivation cohort consisted of 151 patients with hypertension (57 ± 10 years old; 58% males). Patients with replacement myocardial fibrosis (*n* = 55) had a higher systolic blood pressure compared to those without (138 ± 15 vs. 130 ± 13 mmHg, respectively; *P* = 0.001). These patients had higher indexed LV mass (69 ± 22 vs. 52 ± 10 g/m^2^, respectively; *P* < 0.001), and higher measures of reactive interstitial myocardial fibrosis (ECV and native T1; *P* < 0.001 for all; Table 1).

**Table 1:** Characteristics of patients in the derivation cohort.

	**All patients**	**No myocardial fibrosis**	**Myocardial fibrosis**	***P*-value**
	**(*n =* 151)**	**(*n =* 96)**	**(*n =* 55)**	
**Clinical characteristics**				
Age (years)	57 ± 10	57 ± 9	57 ± 12	0.849
Males, *n* (%)	87 (58)	49 (51)	38 (69)	0.031
Diabetes mellitus, *n* (%)	47 (31)	26 (27)	21 (38)	0.156
Height (*m*)	1.65 ± 0.09	1.64 ± 0.08	1.66 ± 0.10	0.186
Weight (kg)	73.7 ± 17.2	71.6 ± 15.6	77.3 ± 19.1	0.048
Body surface area, m^2^	1.80 ± 0.22	1.77 ± 0.20	1.85 ± 0.25	0.053
Taking 1 anti-hypertensive medication	76 (50)	50 (52)	26 (47)	0.246
Taking 2 anti-hypertensive medications	47 (31)	32 (33)	15 (27)	
Taking 3 anti-hypertensive medications	28 (19)	14 (15)	14 (26)	
Systolic blood pressure (mmHg)	133 ± 14	130 ± 13	138 ± 15	0.001
Diastolic blood pressure (mmHg)	80 ± 10	79 ± 8	83 ± 11	0.013
**Echocardiography characteristics**				
Echocardiographic LV GLS (%)	−17.4 ± 3.4	−18.5 ± 2.6	−15.4 ± 3.7	<0.001
**Cardiovascular magnetic resonance characteristics**				
Indexed LV mass (g/m^2^)	58 ± 18	52 ± 10	69 ± 22	<0.001
Indexed LV EDV (mL/m^2^)	75 ± 15	72 ± 13	78 ± 17	0.019
Indexed LV ESV (mL/m^2^)	31 ± 11	29 ± 7	35 ± 15	0.005
Indexed LV SV (mL/m^2^)	43 ± 9	44 ± 8	43 ± 9	0.759
LV ejection fraction (%)	60 ± 7	60 ± 7	61 ± 11	0.538
Native T1 (ms)	1,027 ± 30	1,019 ± 26	1,041 ± 31	<0.001
ECV (%)	26.2 ± 2.9	25.4 ± 2.5	27.4 ± 3.1	<0.001

*GLS, global longitudinal strain; LV, left ventricular; EDV, end-diastolic volume; ESV, end-systolic volume; SV, stroke volume; ECV, extracellular volume fraction*.

**Figure 1:**
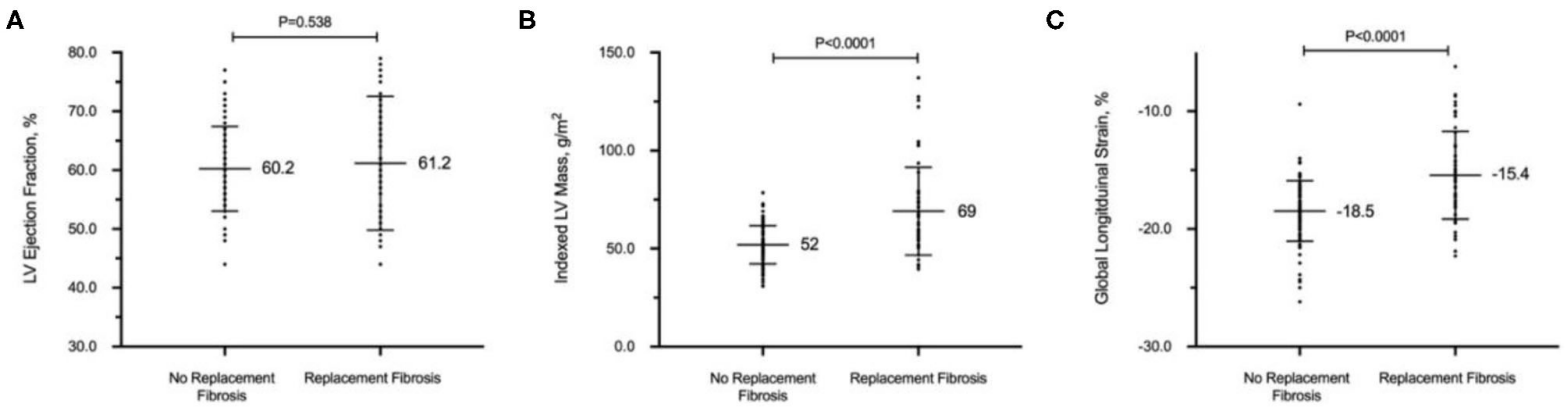
Replacement myocardial fibrosis on cardiovascular magnetic resonance in patients with hypertension. Despite similar left ventricular (LV) ejection fraction **(A)**, patients with replacement myocardial fibrosis had increased indexed LV mass **(B)** and worse global longitudinal strain on echocardiography **(C)**. All values in mean and standard deviation.

LV GLS demonstrated a moderately strong correlation with indexed LV mass (*r* = 0.62; *P* < 0.001) but a weaker correlation with native T1 (*r* = 0.34; *P* < 0.001) and ECV (*r* = 0.15; *P* = 0.071). Despite similar LVEF, patients with replacement myocardial fibrosis demonstrated impaired LV GLS on echocardiography (−15.4 ± 3.7 vs. −18.5 ± 2.6%, respectively; *P* < 0.001, Figure 1). Indexed LV mass (standardized coefficient = 0.48; *P* < 0.001) and replacement myocardial fibrosis (standardized coefficient = 0.18; *P* = 0.013) were independently associated with LV GLS, after adjusting for age, sex and systolic blood pressure.

### Establishing GLS Thresholds of Myocardial Fibrosis From the Derivation Cohort

In the analysis to determine LV GLS thresholds of replacement myocardial fibrosis, LV GLS was the only variable associated with replacement myocardial fibrosis (OR 1.41; 95% confidence interval 1.22–1.62; *P* < 0.001). Age, sex and systolic blood pressure were not retained in the regression model. LV GLS demonstrated high discrimination (AUC 0.74; 95% confidence interval 0.66–0.83; *P* < 0.001) and calibration (Hosmer-Lemeshow χ^2^= 6.37; *P* = 0.605) for the presence of replacement myocardial fibrosis. Based on the ROC analysis, a LV GLS threshold of −21.0% was associated with 95% sensitivity to rule out replacement myocardial fibrosis, while a LV GLS threshold of −15.0% was associated with 95% specificity to rule in replacement myocardial fibrosis.

In the derivation cohort, 14 patients had LV GLS < −21.0% (better strain) and were considered as low-risk. Conversely, 28 patients had LV GLS more than −15.0% (worse strain) and were classified as high-risk. The other patients with LV GLS values between −15.0 and −21.0% were considered at intermediate risk. High-risk patients were predominantly males (*n* = 22, 79%) and had the highest systolic blood pressure compared to the other risk groups (*P* < 0.001; Table 2). Across the risk groups of LV GLS, there was a step-wise increase in LV mass (Table 2) that remained significant after adjusting for the effects of age, sex and systolic blood pressure. High-risk patients had the highest proportion of replacement myocardial fibrosis. As a CMR marker of reactive interstitial fibrosis, native T1 (but not ECV) was the highest in the group of patients with the worst LV GLS (Table 2).

**Table 2:** Characteristics of patients in the derivation cohort, stratified by global longitudinal strain.

	**Less than 21.0%**	**−21.0 to −15.0%**	**More than −15.0%**	***P*-Value**
	**(*n =* 14)**	**(*n =* 109)**	**(*n =* 28)**	
**Clinical characteristics**				
Age (years)	57 ± 6	57 ± 10	56 ± 13	0.974
Males, *n* (%)	2 (14)	63 (58)	22 (79)	<0.001
Diabetes mellitus, *n* (%)	3 (21)	33 (30)	11 (39)	0.468
Height (m)	1.59 ± 0.07	1.65 ± 0.09	1.66 ± 0.09	0.057
Weight (kg)	63.9 ± 9.2	72.5 ± 16.2	83.2 ± 20.0	0.001
Body surface area, m^2^	1.66 ± 0.14	1.79 ± 0.22	1.90 ± 0.22	0.002
Systolic blood pressure (mmHg)	122 ± 11	132 ± 13	143 ± 17	<0.001
Diastolic blood pressure (mmHg)	74 ± 5	80 ± 9	84 ± 12	0.004
**Cardiovascular magnetic resonance characteristics**				
Indexed LV mass (g/m^2^)	44 ± 8	55 ± 10	78 ± 27	<0.001
Indexed LV EDV (mL/m^2^)	64 ± 10	74 ± 12	83 ± 22	<0.001
Indexed LV ESV (mL/m^2^)	23 ± 5	30 ± 7	39 ± 19	<0.001
Indexed LV SV (mL/m^2^)	41 ± 8	44 ± 8	44 ± 11	0.579
LV ejection fraction (%)	64 ± 6	59 ± 7	55 ± 12	0.001
Non-ischemic LGE, n (%)	2 (14)	30 (28)	23 (82)	<0.001
Native T1 (ms)	1,016 ± 22	1,024 ± 27	1,043 ± 37	0.003
ECV (%)	25.2 ± 2.3	26.1 ± 2.6	26.9 ± 4.0	0.191

*See Table 1*.

### Association Between LV GLS and Adverse Cardiovascular Events in the Outcome Cohort

The outcome cohort consisted of 261 patients with moderate to severe AS with preserved LVEF (71 ± 12 years old; 58% males; Vm = 3.8 ± 0.7 m/s; Table 3). LV GLS had a modest association with AS severity based on indexed AVA (*r* = −0.26; *P* < 0.001) and hemodynamics (Vm: *r* = 0.22; *P* < 0.001). Using the LV GLS thresholds established in the derivation cohort, 11.9% (*n* = 31) of the patients were classified as low-risk and 46.4% (*n* = 121) were classified as high-risk. High-risk patients with AS had the most severe AS disease, highest E/e′ ratio and lowest LVEF compared to the other groups (Table 3). Of note, blood pressures were similar across the risk groups (*P* = 0.674).

**Table 3:** Characteristics of patients in the outcome cohort, stratified by global longitudinal strain.

	**All patients**	**Less than −21.0%**	**−21.0 to −15.0%**	**More than −15.0%**	***P*-value**
	**(*n =* 261)**	**(*n =* 31)**	**(*n =* 109)**	**(*n =* 121)**	
**Clinical characteristics**					
Age (years)	71 ± 12	74 ± 10	69 ± 12	73 ± 12	0.033
Males, *n* (%)	151 (58)	9 (29)	58 (53)	84 (69)	<0.001
Diabetes mellitus, *n* (%)	90 (35)	9 (29)	40 (37)	41 (34)	0.718
Hypertension, n (%)	197 (76)	24 (77)	77 (71)	96 (79)	0.299
Hyperlipidemia, *n* (%)	161 (62)	21 (68)	72 (66)	68 (56)	0.234
Coronary artery disease, *n* (%)	74 (28)	10 (32)	17 (16)	47 (39)	<0.001
Height (m)	1.63 ± 0.11	1.62 ± 0.12	1.59 ± 0.10	1.67 ± 0.10	<0.001
Weight (kg)	69 ± 16	66 ± 14	63 ± 12	75 ± 17	<0.001
Body surface area, m^2^	1.74 ± 0.24	1.70 ± 0.23	1.65 ± 0.20	1.84 ± 0.23	<0.001
Systolic blood pressure (mmHg)	144 ± 22	146 ± 27	144 ± 21	143 ± 22	0.674
Diastolic blood pressure (mmHg)	74 ± 11	71 ± 13	73 ± 10	75 ± 11	0.059
**Echocardiography**					
Aortic valve area (cm^2^)	0.87 ± 0.23	0.93 ± 0.20	0.94 ± 0.23	0.80 ± 0.22	<0.001
Indexed aortic valve area (cm^2^/m^2^)	0.51 ± 0.14	0.56 ± 0.14	0.57 ± 0.13	0.44 ± 0.12	<0.001
Mean pressure gradient (mmHg)	42 ± 17	38 ± 15	39 ± 16	45 ± 17	0.018
Peak aortic velocity (m/s)	3.8 ± 0.7	3.7 ± 0.9	3.8 ± 0.7	3.9 ± 0.7	0.368
LV ejection fraction, biplane Simpson (%)	63 ± 6	66 ± 7	66 ± 4	59 ± 6	<0.001
Indexed LV mass (g/m^2^)	123 ± 35	107 ± 24	113 ± 24	136 ± 40	<0.001
E/e′ ratio	15.8 ± 7.4	14.7 ± 7.3	14.8 ± 6.6	17.0 ± 7.9	0.063
LV GLS (%)	−16.1 ± 3.6	−22.3 ± 1.4	−17.7 ± 1.6	−13.0 ± 1.8	<0.001

*See Table 1*.

**Figure 2:**
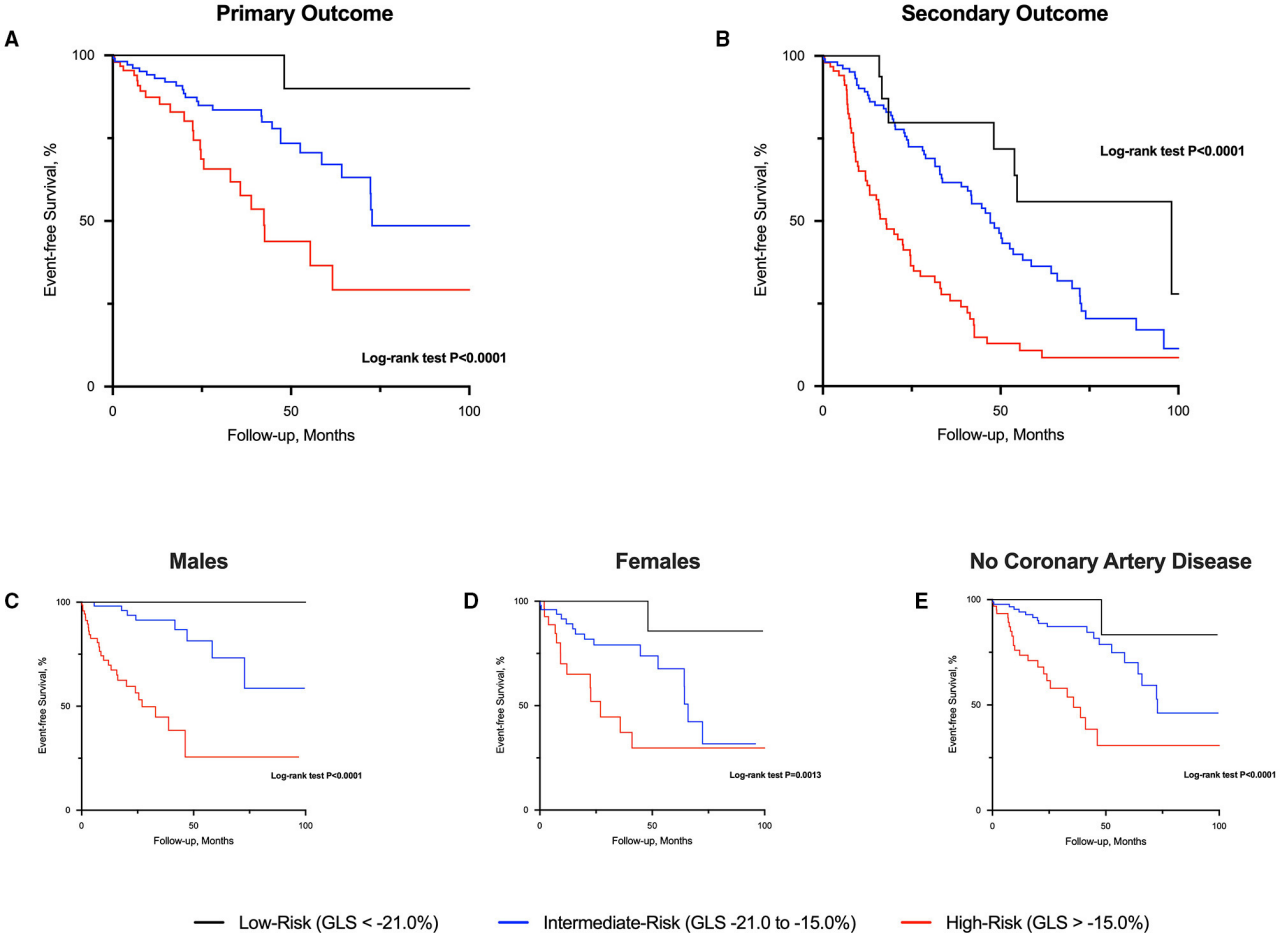
Event-free survival curves in patients with aortic stenosis risk-stratified by global longitudinal strain. High-risk patients with aortic stenosis (global longitudinal strain; GLS > −15.0%) had worse prognosis compared to those at intermediate and low-risk (GLS < −21.0%) for both primary **(A)** and secondary **(B)** outcome. Similar prognostic findings were observed regardless of sex **(C,D)** and excluding those with coronary artery disease **(E)**.

There were 47 primary cardiovascular events (cardiovascular deaths, *n* = 10; heart failure hospitalization, *n* = 23; myocardial infarction, *n* = 14) that occurred during 16 [3.3, 42.2] months of follow-up (569.3 patient-years; 8.3 events/100 patient-years). Only 1 event occurred in low-risk patients. Conversely, 23 events occurred in those at high-risk and the remaining 23 events in those at intermediate-risk (log-rank *P* < 0.001; Figure 2). For the secondary outcome, a total of 143 patients experienced an event: heart failure, *n* = 23; myocardial infarction, *n* = 14; AVR, *n* = 77; and all-cause deaths, *n* = 29. Patients classified as high-risk experienced more secondary events compared to those at intermediate- and low-risk (Figure 2). Similar findings were observed when analyses were sex-stratified and after excluding those patients with coronary artery disease (Figure 2).

**Figure 3:**
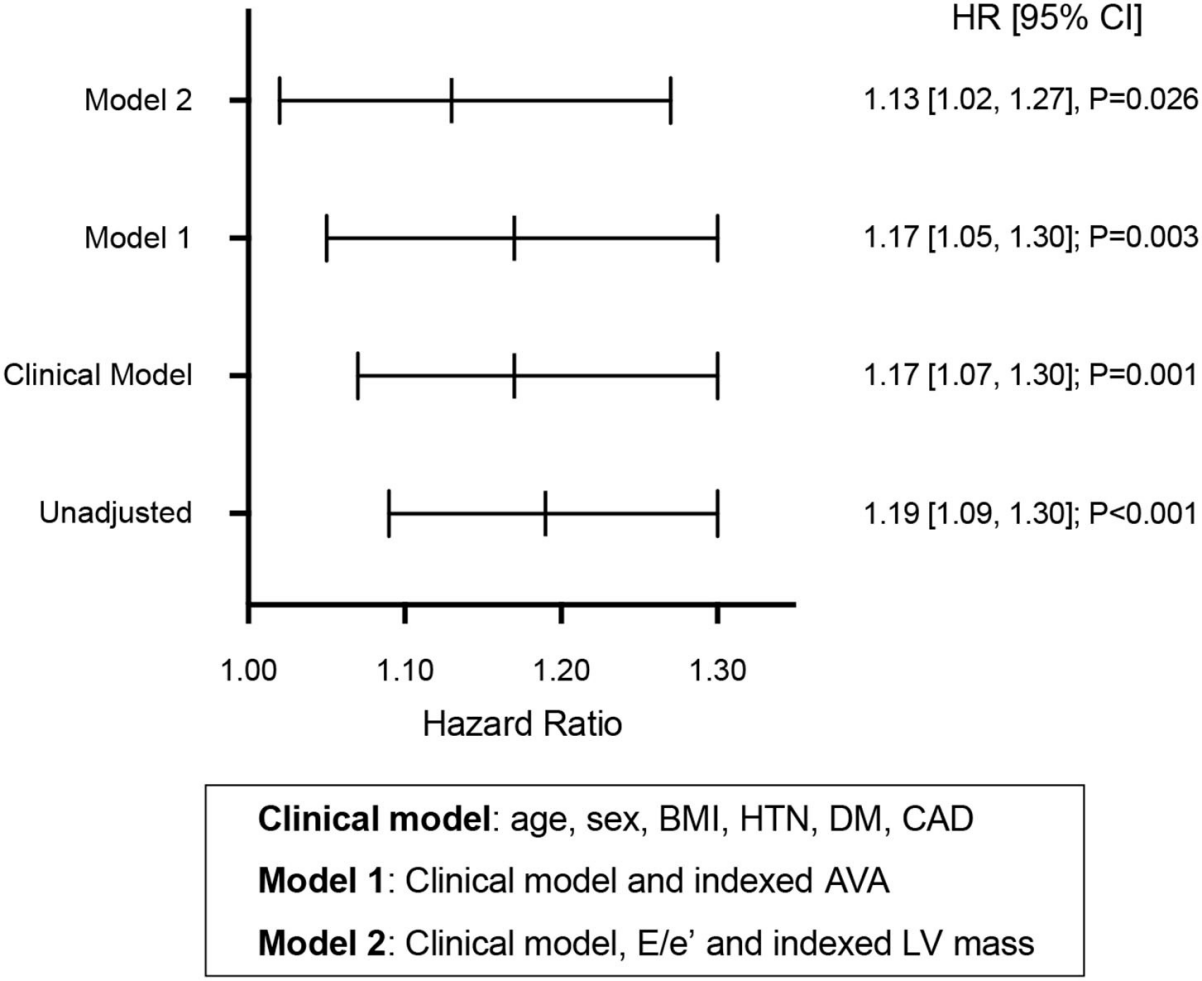
Prognostic value of global longitudinal strain in aortic stenosis. Hazard ratios (HR) predicting time to adverse cardiovascular events for global longitudinal strain in unadjusted and adjusted cox models. BMI, body mass index; HTN, history of hypertension; DM, history of diabetes mellitus; CAD, history of coronary artery disease; AVA, aortic valve area; LV, left ventricular.

LV GLS was independently associated with adverse cardiovascular events in separate models after adjusting for the potential confounding effects of clinical risk factors (age, sex, body mass index, history of hypertension, diabetes and coronary artery disease), AS severity, LV mass and E/e′ ratio (Figure 3).

## Discussion

The current study demonstrates that LV mass and replacement myocardial fibrosis on CMR were independently associated with echocardiographic LV GLS; while CMR measures of reactive interstitial fibrosis had weaker correlations with LV GLS. LV GLS thresholds of replacement myocardial fibrosis risk-stratified patients with AS. Despite absent/minimal symptoms and preserved LVEF, patients with moderate or severe AS deemed at high risk (LV GLS > −15.0%) experienced increased cardiovascular events compared to those at intermediate- (LV GLS −15.0 to −21.0%) and low-risk (LV GLS < −21.0%) over medium term follow-up. LV GLS was independently associated with adverse cardiovascular outcomes after adjusting for the effects of potential confounders (Graphical Abstract).

In the pathophysiology of LVH, increased wall thickness and decreased chamber size initially normalize elevated wall stress. Collagen accumulates in a diffuse pattern within the interstitium and progresses to replacement fibrosis that corresponds to regions of cellular necrosis/apoptosis (3, 24). Therefore, changes affecting the interstitium and intrinsic myocardial contractility can influence myocardial deformation (25). In our study, LV GLS is independently associated with LV mass and to a lesser extent, replacement fibrosis. This observation can partly be explained by the technique of LV GLS by speckle tracking. The echocardiographic technique of assessing GLS by speckle tracking relies on tracking the displacement of speckles generated by the interaction between ultrasound waves and *myocardial fibers* (26). A recent study in young pigs demonstrated that LVH remodeling (as opposed to myocardial fibrosis) contributed more to reduced LV GLS after 19 weeks of aortic banding (27). This, along with our current study, would suggest LV GLS is mediated to a greater extent by increased LV mass; and interstitial abnormalities (myocardial fibrosis) affect LV GLS as a function of reduced intrinsic contractility only when LVH is more advanced. Of relevance, the myocardial architecture is a complex array of fibers organized in layers: subendocardial layer oriented longitudinally (more susceptible to wall stress and reduced perfusion) and subepicardial layer oriented more obliquely (28). Recent evidence demonstrated subendocardial GLS as a marker of disease severity, symptoms and prognosis (29, 30). The association between specific layer GLS, myocardial fibrosis and prognosis is of topical interest and warrants further investigation.

There is increasing recognition that AS is a condition that not only affects the aortic valve but also the myocardium (31, 32). The magnitude of LVH in response to AS is heterogeneous and this may partially account for the weak association between AS severity and LV mass (33, 34). Similarly, the association between LV GLS and AS severity has not been consistently demonstrated (35–37). In our study, we did not observe a strong correlation between LV GLS and measures of AS severity. An integrated multimodality approach of assessing the valve and myocardium is therefore necessary in the optimal management of patients with AS, particularly before the onset of symptoms and development of heart failure (38).

Conventional and novel CMR techniques have increased our ability to characterize the myocardium (2). The limited availability and high costs of CMR may make routine surveillance impractical and not cost-effective. Consequently, markers of myocardial fibrosis, particularly those with prognostic value are potentially attractive. In our previous studies, we have developed a clinical risk score (consisting of age, sex, AS severity, high-sensitive cardiac troponin I concentration and electrocardiographic strain pattern) based on its mechanistic association with replacement myocardial fibrosis on CMR (12–14). In that study, asymptomatic patients with moderate to severe AS and a high-risk score for myocardial fibrosis have the worst outcomes compared to patients at intermediate- and low-risk (14). In the current study, we have validated the prognostic potential of echocardiographic LV GLS, another marker associated with replacement myocardial fibrosis. A highly specific LV GLS value for replacement fibrosis (GLS > −15.0%) predicts worse cardiovascular outcomes compared to a threshold that is highly sensitive for replacement fibrosis (GLS < −21.0%). These GLS values are also consistent with data-driven thresholds from studies in moderate disease and asymptomatic severe AS (39, 40). Our studies highlight the novel approach of risk-stratifying patients based on the association with a pathophysiologically relevant substrate (myocardial fibrosis) of cardiac failure.

In our study, 14 patients experienced myocardial infarction as the primary outcome. Unlike heart failure, the association between myocardial fibrosis and myocardial infarction may be less obvious. Coronary flow reserve (CFR) is impaired in patients with aortic stenosis, even in the absence of epicardial coronary artery disease (41). Although the exact mechanisms are unknown, increased myocardial wall stress and left ventricular hypertrophy are associated with impaired CFR (42). Impaired CFR results in myocardial ischemia that is manifested clinically as angina and myocardial infarction. In a recent study, we have demonstrated elevated high sensitivity cardiac troponin I concentrations in patients with aortic stenosis and replacement myocardial fibrosis on CMR (12). These findings support a mechanistic association between myocardial fibrosis and ischemia.

### Clinical Implications

Risk stratification in AS with either echocardiographic LV GLS, the clinical risk score or other approaches would need to be guided by local expertise and availability of resources. LV GLS, as a marker of replacement fibrosis, has incremental prognostic value over conventional prognostic markers such as LVEF, AS severity and E/e′. These observations may impact future management of AS. Potentially, asymptomatic patients with moderate to severe AS and preserved LVEF can be further risk-stratified using echocardiographic LV GLS. Patients at intermediate risk may benefit from further risk stratification with CMR (for replacement and diffuse myocardial fibrosis), computed tomography aortic valve calcium score and/or exercise stress testing. Whether these patients identified to be at high-risk would benefit from early AVR would need to be guided by future trials.

### Study Limitations

Myocardial deformation is affected by co-existing cardiac conditions. As this study attempts to validate LV GLS as a novel marker of replacement myocardial fibrosis, we have carefully excluded cardiac conditions that may confound the assessment of LV GLS in the derivation cohort. The prognostic value of these LV GLS thresholds on the individual endpoint would need to be verified in other more heterogeneous cohorts of asymptomatic patients with significant AS. These thresholds will also require further validation with different speckle tracking software, imaging modalities (such as CMR) and techniques of assessing GLS [such as the novel 3D speckle tracking (43)]. GLS was not assessed in patients after AVR that should be investigated in future studies.

## Conclusion

Echocardiographic LV GLS has an independent association with replacement myocardial fibrosis and LV mass on CMR. Asymptomatic patients with significant AS and LV GLS thresholds specific for myocardial fibrosis (GLS > −15.0%) are at high-risk of adverse cardiovascular outcomes. Conversely, those at low-risk (GLS < −21.0%) have very favorable prognosis. LV GLS thresholds of replacement fibrosis is a novel approach to risk-stratify patients with moderate to severe AS and preserved LVEF who may benefit from early AVR.

## Data Availability Statement

The raw data supporting the conclusions of this article will be made available by the authors, without undue reservation.

## Ethics Statement

The studies involving human participants were reviewed and approved by Singhealth CIRB and National Healthcare Group DSRB. The patients/participants provided their written informed consent to participate in this study.

## Author Contributions

T-TL, WH, L-HL, and CC conceived the study idea. T-TL and WH contributed in data analysis, manuscript drafting, and preparation. BA, JAB, and D-FT recruited the patients, collected study/imaging data, and curated the data in the Singapore derivation cohort. SE, HT, ET, JY, QY, EL, PY, SC, PG, LL, HO, AR, and L-HL recruited aortic stenosis patients in the multi-center study, collected and curated the study data in their respective sites in the Singapore validation cohort. WS, YO, LG, and JL assisted in the data/imaging analysis of patients in the Singapore validation cohort. GS, VD, and JJB collected, analyzed, and curated the clinical and imaging data in the Leiden validation cohort. L-HL and CC contributed in supervising the writing and interpretation of the data. All authors have made critical feedback and contributed to the final manuscript.

## Funding

This study was funded by the National Medical Research Council, Singapore (NMRC/CG/NUHCS/2010, NMRC/CG12Aug14, NMRC/CGAug16/C006, MOH-CSAINV17nov-0002), and the Charles Toh Cardiovascular Fellowship Fund.

## Conflict of Interest

The department of Cardiology at Leiden University receives unrestricted research grants from Abbott Vascular, Bayer, Biotronik, BIoventrix, Boston Scientific, Edwards Lifesciences, GE Healthcare, Ionis and Medtronic. VD received speaker fees from Abbott Vascular, Edwards Lifesciences, GE Healthcare, MSD, Medtronic and Novartis. JJB received speaker fees from Abbott Vascular. The remaining authors declare that the research was conducted in the absence of any commercial or financial relationships that could be construed as a potential conflict of interest.

## Publisher's Note

All claims expressed in this article are solely those of the authors and do not necessarily represent those of their affiliated organizations, or those of the publisher, the editors and the reviewers. Any product that may be evaluated in this article, or claim that may be made by its manufacturer, is not guaranteed or endorsed by the publisher.

## Abbreviations

AS: Aortic stenosis
AVA: Aortic valve area
AVR: Aortic valve replacement
CMR: Cardiovascular magnetic resonance
ECV: Extracellular volume fraction
GLS: Global longitudinal strain
LVEF: Left ventricular ejection fraction
LVH: Left ventricular hypertrophy
MPG: Mean pressure gradient
NYHA: New York Heart Association
Vm: Peak aortic velocity.
